# Implementing early mobilisation after knee or hip arthroplasty to reduce length of stay: a quality improvement study with embedded qualitative component

**DOI:** 10.1186/s12891-020-03780-7

**Published:** 2020-11-20

**Authors:** Happy Chua, Bernadette Brady, Melissa Farrugia, Natalie Pavlovic, Shaniya Ogul, Danella Hackett, Dimyana Farag, Anthony Wan, Sam Adie, Leeanne Gray, Michelle Nazar, Wei Xuan, Richard M. Walker, Ian A. Harris, Justine M. Naylor

**Affiliations:** 1 South Western Sydney Local Health District, Locked Bag 7103, Liverpool, NSW BC 1871 Australia; 2 St George and Sutherland Clinical School, St George Hospital, Short St, Kogarah, NSW 2217 Australia; 3 Ingham Institute Applied Medical Research, 2 Campbell St, Liverpool, NSW 2170 Australia; 4 Whitlam Orthopaedic Research Centre, Ingham Institute Applied Medical Research, 2 Campbell St, Liverpool, NSW 2170 Australia; 5 South Western Sydney Clinical School UNSW Sydney, Locked bag 7103, Liverpool BC, NSW Australia; 6 South West Clinical School UNSW, Locked bag 7103, Liverpool BC, NSW Australia

**Keywords:** Arthroplasty, Arthroplasty, knee, Arthroplasty, hip, Early mobilisation, Fast track

## Abstract

**Background:**

Models of care for managing total knee or hip arthroplasty (TKA, THA) incorporating early mobilisation are associated with shorter acute length-of-stay (LOS). Few studies have examined the effect of implementing early mobilisation in isolation, however. This study aimed to determine if an accelerated mobilisation protocol implemented in isolation is associated with a reduced LOS without undermining care.

**Method:**

A Before-After (quasi-experimental) study was used. Standard practice pre-implementation of the new protocol was physiotherapist-led mobilisation once per day commencing on post-operative Day 1 (Before phase). The new protocol (After phase) aimed to mobilise patients four times by end of Day 2 including an attempt to commence on Day 0; physiotherapy weekend coverage was necessarily increased. Poisson regression modelling was used to determine associations between study period and LOS. Additional outcomes to 12 weeks post-surgery were monitored to identify unintended consequences of the new protocol. Time to first mobilisation (hours) and proportion mobilising Day 0 were monitored to assess protocol compliance. An embedded qualitative component captured staff perspectives of the new protocol.

**Results:**

Five hundred twenty consecutive patients (*n* = 278, Before; *n* = 242, After) were included. The new protocol was associated with no change in unadjusted LOS, a small reduction in adjusted LOS (8.1%, *p* = 0.046), a reduction in time to first mobilisation (28.5 (10.8) vs 22.6 (8.1) hrs, *p* < 0.001), and an increase in the proportion mobilising Day 0 (0 vs 7%, *p* < 0.001). Greater improvements were curtailed by an unexpected decrease in physiotherapy staffing (After phase). There were no significant changes to the rates of complications or readmissions, joint-specific pain and function scores or health-related quality of life to 12 weeks post-surgery. Qualitative findings of 11 multidisciplinary team members highlighted the importance of morning surgery, staffing, and well-defined roles.

**Conclusion:**

Small reductions in LOS are possible utilising an early mobilisation protocol in isolation after TKA or THA although staff burden is increased likely undermining both sustainability and the magnitude of the change. Simultaneous incorporation of other changes within the pathway would likely secure larger reductions in LOS.

**Supplementary Information:**

The online version contains supplementary material available at 10.1186/s12891-020-03780-7.

## Background

Osteoarthritis is the most common form of arthritis with 1 in 11 Australians affected in 2017–18 [1]. Hip or knee arthroplasty is often recommended for those patients unresponsive to medication and exercise and is very effective in restoring quality of life [2]. The Australian Institute of Health and Welfare recorded a 38% rise in the rate of total knee arthroplasty (TKA) and a 40% rise in total hip arthroplasty (THA) from 2005 to 06 to 2016–17, with the increasing demand for such surgery placing pressure on already stretched health resources and leading to patients waiting longer for surgery [1].

Earlier discharge home from hospital following arthroplasty and other major surgeries decreases care costs [3–5] and increases capacity in a stretched public health environment. Early mobilisation – variably defined as mobilisation or getting out of bed on the day of surgery or within 24 h of surgery [6] – has been incorporated into models of care as a strategy to decrease acute-care length of stay (LOS) [3, 6–19].

Despite gains to service efficiency, mobilising patients as early as the day of surgery has been observed to be an uncommon practice in Australia [20]. It is unclear why this evidence - practice gap exists and there may be several factors contributing to or explaining the divide. Trials where LOS has been successfully reduced have included early mobilisation as one part of multiple changes to the pathway [3, 5, 14–17], included only those undergoing unicompartmental knee arthroplasty [7, 8], excluded patients with complications or co-morbidities [7, 21], or mandated discharge at 24 h post-operatively regardless of the level of mobility [7]. Thus, it is unclear if early mobilisation in isolation is effective, and if so, if it can be attempted in all patients or only a subset. Services may also have limited capacity to both review and overhaul local models of service delivery as new evidence emerges, thus, there may be a delay in evidence translation. Few studies have examined the effect of implementing an early mobilisation protocol within the confines of existing resources, thus, it is unclear how readily the practice can be adopted without burden on staff or other services. With these explanations in mind, determining if a simple adjustment alone to the mobilisation protocol applied across all patients can achieve a reduction in LOS would be useful and aid broader translation of early mobilisation approaches.

Data from our registry [22] indicated that the LOS for patients undergoing TKA and THA in our centre (~ 5.5 days) where an early mobilisation protocol had not been implemented was comparatively long (3.5 days). This quality improvement study aimed to determine if an accelerated mobilisation protocol implemented in a high-volume, specialist arthroplasty centre in the absence of other major changes and using the same or minimally enhanced resources is associated with a reduced LOS without undermining care such as increasing adverse event or readmission rates.

## Methods

### Setting, design and and ethical approval

The study was conducted at a high-volume (> 600 TKA or THA procedures annually) arthroplasty centre in South West Sydney. A mixed-methods approach was used comprising two distinct component phases. The first phase was a quasi-experimental Before-After implementation study, while the second phase was a qualitative component comprising semi-structured interviews of key members of the multidisciplinary team (MDT). The qualitative methods aimed to explore staff perceptions and experiences related specifically to the implementation of the early mobilisation protocol. We deemed this component essential given that adopting recommended practices in the context of high volume surgery and minimal resource enhancement may be relevant to the success of the strategy, and staff perceptions may expose this.

Ethics approval was granted as a low and negligible risk study by the Institution’s Human Research Ethics Committee. For phase one, participants provided informed, verbal consent including an opt-out option for those wishing not to be included in the post-discharge follow-up. For phase two, written informed consent was obtained from all participants.

### Identification and management of the problem

As described earlier [22], our hospital had longer mean (5.5) and median (4) stays over 2017–18 compared to the LOS reported by other hospitals (mean 3.5, median 3). Whilst the registry data were unable to pin-point the exact cause for the differences in LOS, it was known generally that the hospitals with the lower LOS had introduced early mobilisation protocols.

On review of published research regarding early mobilisation, the studied interventions varied markedly [3, 5–19]. Much of the research was arguably outdated, as there were multiple concurrent changes were made to existing protocols, and there were mixed definitions of early mobilisation. It was difficult to identify the specific intervention that reduced LOS or improved mobility outcomes. Instead, we chose to change one aspect of the intervention in order to evaluate the effect of early mobilisation on LOS. The Before-After design was chosen in preference to a parallel randomised trial as applying different moblisation protocols to individuals concurrently within the same ward was not practical.

A MDT was formed comprising the following disciplines: orthopaedic surgery, anaesthetics, physiotherapy, occupational therapy, nursing, social work, acute pain management, and, research. The team met regularly to develop the intervention and the research protocol, including the identification of outcome measures to be collected and the standardisation of key practices e.g. pain management, anaesthetic and other ward-based protocols (Table 1).

**Table 1:** Peri-operative and post-operative protocols for the historical and intervention periods

	Before cohort	After cohort
Spinal anaesthetic	Preferred approach	Preferred approach
Adductor canal block	Standard protocol unless contraindicated in TKA	Standard protocol unless contraindicated in TKA
Tranexamic Acid (intravenous)	Standard protocol unless contraindications	Standard protocol unless contraindications
Patient controlled anaesthesia	Optional	Optional
Multi-modal analgesia	Standard protocol	Standard protocol
In-dwelling catheter	Standard protocol	Standard protocol
Use of tourniquet (TKA only)	Standard protocol	Standard protocol
Medial parapatellar approach (TKA)	Standard	Standard
Posterolateral approach (THA)	Standard	Standard
Mobilisation Day 0	Not performed	Routine attempt
Mobilisation Day 1	Once	Twice
Mobilisation Day 2	Once	Twice

Legend: *TKA* total knee arthroplasty, *THA* total hip arthroplasty

At the time of study commencement, the standard allied health ward practice was for patients to commence mobilisation day 1 postoperatively and receive physiotherapy once per day until discharge. Occupational therapy intervention started when the patient commenced on crutches or their baseline mobility aid, such as a 4-wheeled walking frame. Social work intervention was provided on a referral basis only.

After review of the Before phase, it was apparent that the availability of nursing and physiotherapy staff, and the timing of patients’ return to ward after theatre were potential barriers to achieving the planned protocol. The planned intervention required mobilisation on the day of surgery (Day 0) to be attempted when possible and a target of four occasions of mobilisation by the physiotherapist, or by the nurse in their absence, by the end of Day 2 post- operatively. For both the Before and After phases, successful mobilisation was defined as standing and marching on the spot and/or walking forward. Transfers from bed-to-chair were not considered mobilisation. Given the concern about staffing, a temporary increase in weekend physiotherapy coverage was provided during the intervention period and the hours of physiotherapy coverage during the week were staggered enabling coverage by 1 h into the evening. In addition, nursing staff supported the early mobilisation protocol by sitting patients out of bed earlier and assisting with mobilisation once the patient had been mobilised by the physiotherapist.

### Phase one: participant screening and inclusion

All consecutive patients undergoing primary THA or TKA were eligible for inclusion in the study. Patients waitlisted for surgery were provided with written information about the study. Patients were advised that a quality improvement study was underway to assess and review current practices, and that they would be contacted by researchers after discharge by telephone. Patients were able to verbally opt-out of the telephone follow-up at the time of their preadmission visit. Thus, those who opted-out were excluded from follow-up but details about their acute hospital stay were included.

### Phase one: data extraction, assessment procedures and outcomes

Trained research officers extracted data from paper-based and electronic medical records using study proformas, and obtained data from patients via telephone follow-up at 4- and 12-weeks post-operatively.

Data collection and extraction included typical demographic, primary diagnosis and comorbidity details. When collected or performed as part of the waitlist management program, patient reported surveys were also extracted (Oxford Knee or Hip Scores [23], EuroQol 5 Dimension score [24]) as well as the timed up-and-go (TUG) test [25]. Numerous primary, secondary and tertiary outcomes were collected (described in detail below).

### Phase one: primary, secondary and tertiary outcomes

The primary outcomes (*n* = 2) were unadjusted and adjusted acute hospital LOS. The ward operated on criteria lead discharge from hospital, and was based on discharge from physiotherapy, discharge from occupational therapy, if the patient had been afebrile for 24 h and if the wound was dry. Secondary outcomes assessed adherence to the new protocol and included time to first successful mobilisation (hrs); proportions (%) successfully mobilising Days 0, 1 and 2; average number of occasions successfully mobilised with the physiotherapist by end Day 2; proportions who achieved the minimum threshold of 4 occasions by end Day 2. Tertiary outcomes were collected to monitor adverse or unintended consequences of the protocol change and included discharge destination, incidence of medical emergency team (MET) calls acutely, day cleared for discharge from physiotherapy, day cleared for discharge from occupational therapy, referral for social support packages (Compacs), occurrence of a complication (acute, 4-week and then 12-week), readmission (to 4 and 12 weeks), adequacy of index joint pain management at 4 and 12-weeks, and Oxford Knee or Hip Score and EuroQol today health score (EQVAS, 0–100 scale) at 12 weeks. Other variables were monitored to demonstrate consistency in care practices over the two study periods e.g. use of spinal anaesthesia, tranexamic acid, and peripheral nerve blocks.

### Phase one: sample size and analysis

We aimed to reduce LOS from 5.5 (sd 3.1) days (2017–18) to approximately 3.5 days. The 2-day reduction represented a moderate-to-large standardized effect size (0.645) using the T-statistic (non-centrality parameter) (http://www.sample-size.net/sample-size-means/), thus a sample of 106 would provide 90% power (at α = 0.05) to enable detection of a 2-day difference if there was one. As we also wanted to determine adjusted LOS controlling for up to 20 covariates, we aimed for a minimum sample size of approximately 500 to maximize the observation:covariate ratio (approximately 25:1). Based on the rate of surgery at the time, it was anticipated that the sample would be achieved over a 9–12 month period.

Descriptive statistics (mean, standard deviation (sd), median, interquartile range (IQR) percentages) were used to describe the two cohorts where appropriate. Independent t-tests, χ2 tests or Fisher’s exact test, and Mann Whitney U Test were also used as appropriate to compare the cohorts. Poisson regression, in the absence of overdispersion, was used to determine whether there were significant associations between the study period and unadjusted and adjusted LOS [26]. Variables included in the adjusted models were identified a priori and informed by prior literature concerning factors affecting LOS [25, 27–29], and the team’s clinical judgement. In addition to ‘study period’, the covariates included those that could or are known to affect LOS or capacity to mobilise - age (years), gender, body mass index, level of comorbidity, admission to intensive care or high-dependency unit, presence of other lower limb or back problems, joint (TKA or THA), unilateral or bilateral, primary diagnosis of osteoarthritis, use of peripheral nerve blocks, the presence of a complication, morning surgery, surgery day of the week, and the need for an interpreter. These variables are defined in Additional file 1. Pre-surgery Oxford scores, EQVAS and TUG were not included in the modelling as these were not systematically collected for every patient.

Compliance data were used to interpret the level of adoption of specific aspects of the early mobilisation protocol (e.g Day 0 mobilisation, number of occasions over first 2 days), thus, would help determine what components were implemented and to what extent.

No adjustments were made for multiple primary and secondary outcomes as the necessary Bonferroni adjustment would decrease the significant *p*-value to 0.004, rendering the detection of any significant change (e.g. improvement in LOS or deterioration in care) unlikely. Rather, we interpret our significant findings in the context of other supportive data. No imputation of missing data was undertaken. The data were collated in Excel and analysed using SAS and SPSS Version 24.

### Phase two: embedded qualitative component

All multidisciplinary staff involved in the operationalisation of the mobilisation program were approached for possible inclusion. Purposive sampling across the relevant disciplines sought to ensure the sample provided diverse insights into the implementation and functioning of the protocol. Face-to-face, semi-structured interviews using a topic guide modelled on previous research exploring early mobilisation were conducted by a physiotherapy researcher experienced in qualitative research methods and not involved in the implementation of the mobilisation protocol. All interviews were audio-recorded and transcribed verbatim. To ensure accuracy of participant reflections on the implementation process, all healthcare providers (HCPs) were given the opportunity to review their transcript for comment or amendment prior to analysis [30]. An iterative approach to coding was commenced following each interview, guided by the primary aim of understanding key issues influencing the implementation of early mobilisation. Successive interviews were then conducted until the team were satisfied a sufficient number of diverse HCPs had been drawn from the available pool and thematic saturation had been reached. Saturation in this context was monitored by the progression of theme identification after successive interviews [31]. Following 10 interviews, the codebook achieved relative stability for key themes relevant to understanding the operationalisation of the new protocol. An additional interview was conducted to verify saturation had been reached, and when no new information emerged, data collection was ceased. Data were analysed using inductive thematic analysis [32], conducted alongside data collection and performed in NVivo 12 software (QSR International). Interviews were initially coded by one researcher, experienced in qualitative analysis. A second researcher coded a random sample of 50% of the data and consistency between the two coders was evaluated using Cohen’s kappa, performed in NVivo. Meetings with the primary researcher and key members of the research team reviewed and discussed the emergence of key themes within the data. Key concepts were grouped into categories paralleling the quantitative outcomes, as well as into standalone themes summarizing HCP perceptions of the operationalisation of the new protocol.

## Results

**Fig. 1:**
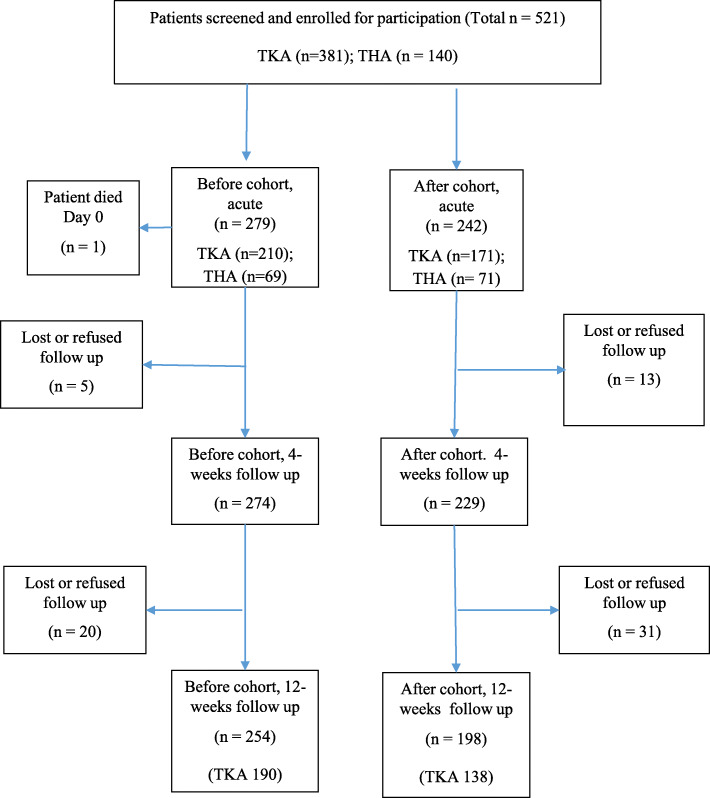
Summary of cohort derivation and retention to 12-weeks post-surgery. TKA, Total Knee Arthroplasty; THA, Total Hip Arthroplasty

Over a 10-month period, 521 people (*n* = 279, Before cohort; *n* = 242, After cohort) underwent primary TKA or THA (*n* = 381 TKA; *n* = 140 THA; *n* = 503 unilateral). One patient died during the acute-care phase; 96.5% (*n* = 503) and 86.8% (*n* = 452) were available for follow-up at 4- and 12-weeks post-surgery (Fig. 1). Compared to those retained, those lost to follow-up (LTFU) at 12 weeks were similar in most key characteristics except for age: female sex [66.7 (LTFU) vs 65.3% (retained), *p* = 0.82], procedure [TKA 76.8 vs 72.6%, *p* = 0.46], pre-surgery BMI [32.3 (6.1) vs 33.0 (6.8), *p* = 0.33], pre-surgery Oxford scores [17.7 (8.8) vs 18.2 (8.2), *p* = 0.69], pre-surgery EQVAS [70 (IQR 29) vs 65 (IQR 30), *p* = 0.76], and age [70.2 (10.2) vs 67.5 (9.5) yr, *p* = 0.04].

Patient characteristics for the two cohorts are summarised in Table 2. The two cohorts were similar in profile for all variables monitored.

**Table 2:** Characteristics of the Historical (Before) and Intervention (After) cohorts

	Before, *n* = 279	After, *n* = 242	*P*-value
Age, yr	67.5 (9.9)	68.3 (9.4)	0.31
Female, n (%)	171 (61.3)	170 (70.2)	0.03
Osteoarthritis, n (%)	266 (95.3)	232 (95.9)	0.77
Total knee arthroplasty n (%)	210 (75.3)	171 (70.7)	0.24
Unilateral, n (%)	270 (96.8)	233 (96.3)	0.76
Time on Wait List, days	325.2 (97.6)	332.5 (87.5)	0.37
Body Mass Index	33.2 (6.8)	32.6 (6.7)	0.26
Body mass index 30+, n (%)	185 (66.3)	151 (62.4)	0.35
**American Society** of **Anesthesiologists** Grade 3 or 4, n (%)	132 (47.5)	114 (47.7)	0.96
Comorbidity, n (%)	265 (95.0)	234 (96.7)	0.33
Hypertension, n (%)	192 (68.8)	164 (67.8)	0.80
Cardiac, n (%)	75 (26.9)	53 (21.9)	0.19
Diabetes, n (%)	58 (20.8)	63 (26)	0.18
Any central nervous system condition, n (%)	24 (8.6)	24 (9.9)	0.61
Other lower limb or back issues, n (%)	147 (57.0)	155 (64.3)	0.09
Chronic respiratory disease, n (%)	49 (17.6)	53 (21.9)	0.21
Renal impairment, n (%)	17 (6.1)	17 (7.0)	0.67
History of venous thromboembolism, n (%)	16 (5.7)	5 (2.1)	**0.03**
Documented mental health condition, n (%)	45 (16.1)	44 (18.2)	0.54
Interpreter required^a^, n (%)	74 (26.5)	81 (33.5)	0.08
Country of birth - English speaking, n (%)	120 (43.0)	102 (42.1)	0.84
Previous knee or hip arthroplasty, n (%)	77 (28.5)	69 (28.6)	0.98
Pre-operative haemoglobin, g/l	138.8 (13.9)	137.3 (15.0)	0.24
Oxford Score^c^	18.0 (8.4)	18.4 (8.1)	0.63
EuroQol Visual analogue score, ‘today’ health,^b^ median (IQR)	66.5 (30)	66.0 (29)	0.90
Timed up-and-go pre-surgery, s ^d^	17.3 (8.5)	16.9 (9.0)	0.68

Values are mean (sd), unless otherwise specified. Chi-Square or Fisher’s exact test for proportions

Legend: *yr* year

^a^A patient who requires an interpreter for verbal or written communication in a language other than English

^b^Missing data *n* = 100

^c^missing data = 99

^d^missing data *n* = 161

For the qualitative phase, 11 healthcare provider interviews were conducted with medical (*n* = 1), orthopaedic (*n* = 1), nursing (*n* = 4) and allied health (*n* = 5) disciplines, each taking a mean of 26 min. The healthcare providers interviewed had on average 9 years of clinical practice (range: 2–22) and the majority were female (*n* = 8). There was high consistency between the two coders, with a kappa of 0.83. Qualitative data were grouped into categories for mobility, length of stay, risk and complications and contributing factors, and are presented alongside these outcomes below. Data relating to the overall conceptualisation of the new model of care are presented as standalone themes below.

Table 3 summarises key intra-operative and acute-care processes and variables for the two time periods. Stability in most of these processes and variables was evident indicating these care processes remained unchanged over the study period and that adherence to ward-based protocols was also similar.

**Table 3:** Intra- and perioperative factors monitored across time

	Before, *n* = 279	After, *n* = 242	*P*-value
Operation time, min	103.0 (25.5)	104.9 (28.6)	0.42
Peripheral nerve block, n (%)	192 (68.8)	164 (67.8)	0.80
Spinal anaesthetic (+/− other), n (%)	242 (86.7)	211 (87.2)	0.88
Patient controlled anaesthesia, n (%)	240 (86.0)	181 (74.8)	**< 0.001**
Use of tranexamic acid, n (%)	250 (89.6)	226 (93.4)	0.13
Use of indwelling catheter, n (%)	278 (99.6)	241 (99.6)	0.92
Tourniquet (knee only), n (%)	205 (95.8)	168 (97.1)	0.49
Donor blood transfusion, n (%)	6 (2.2)	13 (5.5)	0.05
Intensive care/high dependency unit admission, n (%)	16 (5.8))	11 (4.5)	0.54
Day of surgery, n (%)			0.88
Monday	38 (13.6)	27 (11.2)	
Tuesday	80 (28.7)	70 (28.9)	
Wednesday	52 (18.6)	52 (21.5)	
Thursday	33 (11.8)	29 (12.0)	
Friday	76 (27.2)	64 (26.4)	
Morning surgeries (A.M start), n (%)	195 (70.1)	172 (71.1)	0.82

Mobilisation outcomes are summarised in Table 4. In the After phase, the proportion of patients mobilising on Day 0 significantly increased, the time to first mobilisation significantly decreased, and the average number of mobilisation occasions by end Day 2 significantly increased. These statistical changes were mirrored in the qualitative findings whereby all participants reflected on the implementation phase as successful for changing the culture of the early post-operative period to one focused on adopting an active role in recovery: *“I feel like it really has encouraged patients to not adopt a sick role and to engage in therapy sooner” (Participant 10).* Further, participants reflected on a shift in the broad team mindset, wherein *“we’re so used to discharging people day two or three that if anyone got to day four or five, it feels like a long time actually …*. [*yet] that would used to be the usual pathway” (Participant 5).* These changes occurred despite an unexpected reduction in physiotherapy staffing after the first 3-weeks of this phase: *“sick leave that end up being long term … ..then also like a worker’s comp injury that also ended up being quite long term. And given that both of those individuals are on paid leave, to then put someone in to back fill is not really possible because we can’t go over profile” (Participant 7).* The small increase in the proportion mobilising on Day 0 occurred within the first 3-weeks of the intervention phase just prior to the unexpected sick leave. Alongside staffing issues, other reasons for why patients did not mobilise on Day 0 are summarised in Table 4. Commonly reported reasons included ‘return to ward too late’, a finding that was emphasised by participants in interviews as a product of the surgical list organisation. In such cases participants suggested the prioritisation of arthroplasty surgery early in the morning was a necessary pre-requisite for Day 0 mobilisation to occur. The reported mobilisation with nursing staff on Days 0 and 1 was infrequent across both time periods; unexpectedly, significant decreases in mobilisation with nursing staff occurred in the After phase for days 1 and 2. This finding was generally supported by different team members’ accounts of how the responsibility for Day 0 or Day 1 mobilisation was shared across the team during the implementation phase, with most agreeing that despite it being intended to be a shared responsibility (*“not just the physios …*. W*e were expected to do it on our own as well”; Participant 1)*, operationally it appeared to became more physiotherapy-led *“so I just kind of made the decision. I was like, okay, how about just physio take care of day zero and then get nurses to help patients out of bed day one? They were happier with that because they have already done that previously” (Participant 5).*

**Table 4:** Mobilisation statistics

	Before, *n* = 279	After, *n* = 243	*P*-value
Time first mobilised since surgery, hr., mean (sd)	28.5 (10.8)	22.6 (8.1)	< 0.001
Mobilised within 24 h of surgery, n (%)	91 (32.9)	144 (59.5)	< 0.001
Mobilised with physiotherapist successfully Day 0, n (%)	0	16 (6.6)	< 0.001
Patients attempted to mobilise with physiotherapist Day 0, n (%)	0	64 (26)	< 0.001
Number of times mobilised with physiotherapist, Day 0, mean (sd)	0	0.07 (0.25)	< 0.001
Mobilised with physiotherapist Day 1, n (%)	234 (84)	227 (94)	0.001
Number of times mobilised with physiotherapist, Day 1, mean (sd)	0.85 (0.35)	1.68 (0.60)	< 0.001
Mobilised with physiotherapist Day 2, %	259 (93.5)	217 (92.7)	0.732
Number of times mobilised with physiotherapist, Day 2, mean (sd)	0.98 (0.32)	1.53 (0.64)	< 0.001
Total occasions mobilised with physiotherapist by end Day 2, mean (sd)	1.79 (0.56)	3.23 (0.9)	< 0.001
Reached threshold of 4 occasions with physiotherapist, %	0	45	< 0.001
Mobilised with nurse Day 0, n (%)	1 (0.4)	0	1.0
Mobilised with nurse Day 1, n (%)	70 (25.5)	37 (15.4)	0.004
Mobilised with nurse Day 2, n (%)	141 (52)	100 (42.7)	0.037
Reasons for not mobilising Day 0, n (%)^a^			NA
Baseline period	279 (100)	NA	
Return to ward too late	0	76 (33.8)	
Physiotherapy staff unavailable	0	75 (33.3)	
Persistent nerve deficit	0	27 (12)	
Complication e.g. dizziness, Nausea	0	15 (6.7)	
Pain	0	4 (1.8)	
Refused	0	2 (0.9)	
Intensive care or high-dependency unit	0	5 (2.2)	
Other unspecified	0	21 (9.3)	

*NA* not applicable

Legend: *hr.* hour, *e.g.* example

^a^The sample size for “Reasons for not mobilising Day 0” in the After period was based on the 225 responses provided

Table 5 summarises the acute-care outcomes. There was a small, but not statistically significant decrease in unadjusted mean LOS in the After period. There was a significant reduction in the median days cleared for discharge by both the physiotherapist and occupational therapist, consistent with participant accounts that “*I think that it got them independent at the point of discharge quicker, if it was successful” (Participant 1).* The proportion admitted to ICU/HDU remained stable across the two time periods (Table 3) as did the proportions discharged to inpatient rehabilitation, referrals for community social support packages (Compacs) and those experiencing complications or MET calls acutely. The latter was despite a perception among a third of the participants that the early mobilisation was associated with a greater risk of a syncopal event: *“because of the surgery recent, day zero, their BP drops, that’s a major issue. And then they pass out, and the MET call is extra. I feel extra because you get the patients out in day one. I have noticed less, I mean vasovagal can happen day 1, but I think it is lesser to happen on day one, but I think day zero happens more” (Participant 3).*

**Table 5:** Acute outcomes

	Before, *n* = 279	After, *n* = 243	*P*-value
Length of stay, day, mean^b^	4.8 (2.0)	4.6 (2.9)	0.47
Inpatient rehabilitation, n (%)	28 (10.1)	20 (8.3)	0.48
Complications (major or minor), n (%)	50 (17.9)	53 (21.9)	0.26
Medical emergency team calls, n (%)^a^	20 (8.3)	20 (8.3)	0.99
Referred for Compacs, n (%)	25 (9)	17 (7.1)	0.45
Day discharged by physiotherapist, median	4.0 (3,5)	3.0 (2,5)	0.002*
Day discharged by occupational therapist, median	4.0 (3,5)	3.0 (3,5)	0.024*

^a^incomplete reporting

^b^The *p*-value is the unadjusted poisson value

*Mann Whitney U Test

In multiple regression modelling, ‘time period’ (i.e the new mobilisation protocol) was associated with a small, borderline significant reduction in LOS (8.1%, *p* = 0.046) (Table 6). Notably, experiencing a complication, increasing age and admission to intensive care/ high-dependency were the most significantly influential covariates. These findings were consistent with the collective accounts of participants that highlighted while early mobility was the major change targeted with the new protocol, many factors influenced the success of early mobilisation and ultimately length of stay outcomes: “*It felt perhaps like it was a bit hit and miss. So obviously there’s other factors in play that have a big influence on, you know, how quickly someone is able to progress and able to get home, that maybe we can’t impact” (Participant 7).*

**Table 6:** Association between study period and LOS using multiple regression modelling

Parameter	Estimate	Wald 95% Confidence Limits		Wald Chi-Square	*P*-Value
Intervention period (ref Before)^a^	−0.085	−0.169	−0.001	3.97	0.046
Day of surgery (ref Monday)					
Tuesday	0.075	−0.071	0.221	1.02	0.313
Wednesday	0.120	− 0.035	0.275	2.31	0.128
Thursday	0.174	0.004	0.346	4.00	0.046
Friday	0.033	−0.114	0.180	0.19	0.662
Male	0.155	0.063	0.248	10.86	0.001
Total knee arthroplasty	0.009	−0.137	0.155	0.01	0.905
Unilateral procedure	0.251	0.037	0.464	5.30	0.021
Osteoarthritis	0.118	−0.113	0.349	1.00	0.317
Surgery start, morning	0.039	−0.058	0.136	0.61	0.433
ASA 3 or 4	−0.041	−0.134	0.052	0.76	0.384
English-speaking interpreter required	−0.146	−0.237	− 0.055	9.88	0.002
Other lower limb or back issues	−0.057	−0.144	0.029	1.71	0.191
ICU/HDU admission	−0.352	−0.524	− 0.180	16.06	<.0001
Acute complication	−0.389	−0.485	− 0.294	63.91	<.0001
Peripheral nerve block	−0.033	−0.163	0.097	0.25	0.616
Age, yrs	0.012	0.007	0.018	22.27	<.0001
Body mass index, kg/m^2^	0.007	0.001	0.014	4.43	0.035

The incident risk ratio derived from the estimate is 0.92 (0.85 to 1.00). Thus the intervention period was associated with an 8.1% reduction in length of stay

Legend: *ASA* American Society of Anesthesiologists, *ICU* Intensive Care Unit, *HDU* High Dependency Unit, *yrs* years

^a^refer to Before period

Table 7 summarises outcomes at 4- and 12-weeks post-surgery. With one exception (adequacy of pain management), no significant differences were observed in outcomes between the two cohorts.

**Table 7:** Outcomes at 4 and 12-weeks post-surgery

	Before cohort, max *n* = 274	After cohort, max *n* = 229	*P*-Value	Before cohort, max *n* = 254	After cohort, max *n* = 198	*P*-Value
	4-week follow-up			12-week follow-up		
Adequacy of index joint pain management, n (%)			0.487			0.017
Very good	117 (43.8)	94 (42.2)		166 (65.9)	114 (59.4)	
Good, but could be better	128 (48.1)	110 (49.3)		68 (27.0)	73 (38.0)	
Poor	19 (7.1)	19 (8.5)		16 (6.3)	5 (2.6)	
Very poor	3 (1.1)	0		2 (0.8)	0	
Index joint pain (0-10)	3.4 (2.5)	2.9 (2.4)	0.03	1.9 (2.5)	1.9 (2.0)	0.76
Complications (inclusive of acute complications), n (%)	89 (32.5)	74 (32.3)	0.97	93 (36.6)	83 (41.9)	0.25
Re-admissions, n (%)	13 (4.9)	8 (3.6)	0.50	17 (6.7)	19 (9.7)	0.25
Community services used, n (%)	36 (13.5)	27 (12.3)	0.69	Not applicable	Not applicable	
Oxford knee or hip scores, mean (sd)	Not applicable	Not applicable		37.4 (7.7)	36.9 (7.0)	0.46
EuroQol VAS Score for Today health, median (IQR)	Not applicable	Not applicable		80.0 (60, 90)	75 (60, 90)	0.472

### Qualitative thematic results

HCP perceptions of the operationalisation of new mobilisation protocol yielded three independent themes: ‘the person centred within the care’, ‘load-benefit appraisal’ and ‘communication and collaboration’, alongside those supplementing outcomes already reported.

#### The person centred within the care

All participants communicated that not all patients were receptive to the concept of an early moblisation protocol.. As such, patient expectations were cited as key barriers or facilitators to the success of the model. Specifically, participants commented that, those who *“were just positive and wanted to go home … ..just pushed themselves a little bit more” (Participant 3),* while those who” *expect things to be, people to help them, instead of them trying to do it themselves” and ““tend to do more poorly”(Participant 6).* Ethnoculture was cited as a key challenge for navigating expectations, with over two-thirds of participants acknowledging there are inherent cultural differences in how recovery is conceptualised:

*“I guess different cultures will have different expectations about how much the health care provider delivers for them and how much they have to do themselves … .it also changes their perspectives on pain management” (Participant 8).*

Overall, there were mixed views regarding how expectations could be managed and patient engagement improved. Some participantscommunicated a preference for top-down approaches, implying that the system/healthcare providers held the knowledge of what was best for patients and were thus responsible for transferring this knowledge so patients would “*embrace the protocol more readily” (Participant 11)*:

*“We have to educate them and say, look, the point of the surgery is that the more you move, the quicker the progress. We don’t want you to delay the progress. And it’s better that you recover quicker, and progress and go home” (Participant 3).*

While for others, a more collaborative approach was adopted that involved asking *“permission from them” (Participant 4) and* “*negotiating with them about the therapy” (Participant 5)*. A third of participants acknowledged patient negotiation as a complex process that required far more intervention than actions undertaken solely in the post-operative phase:

*“They have the education, but I still don’t think that’s helping their expectations to be honest. Even when I’ve watched patients fill in the surveys right after watching the video sometimes. You know they’re asking me what they should put in and I’m trying to say, no, you need to decide what to put in. And then I’m watching what they put it and it doesn’t match what they’ve just watched. So, I think educating people is different to how they feel about how they’re going to go” (Participant 10).*

#### Load-benefit appraisal

All staff acknowledged that the change in practice was accompanied by an increased workload within the MDT. As such, HCPs appraised the value of increased workload demands against the benefit it yielded for patients, themselves/team, and the facility. Some staff observed that a successful Day 0 mobilisation has flow on effects for the team’s caseload on subsequent days: “*if you manage it, day one becomes really good” (Participant 1).* However most were cautious that an unsuccessful Day 0 attempt could be coupled with additional burdens (MET calls, greater time burden and negative impact to other patients sharing the same room) without the overall benefit: “*Definitely in the second day they’re gonna go slow. And then you find them , like day 3 still with a forearm support frame*” *(Participant 3).* From a facility management point of view, many participants acknowledged the potential benefits: *“If it’s length of stay then from that perspective alone, yes it’s worth it” (Participant 11).* However, without additional resourcing, staff were not satisfied that additional demands and redistribution of caseloads were worth the flow on effects to others within the facility or to the team themselves:

*“it wasn’t within normal resources. Someone else has to be bumped off the list to be able to fit an extra session and if they’re not getting good results from that, how do you justify taking that benefit away from somebody else?” (Participant 7).*

“*when we’re getting out the [arthroplasty] patients sooner, there are medical patients coming in … .and they are more complex. You know, the joints at least, we have a protocol, we know what to expect” (Participant 10).*

#### Communication and collaboration

All participants acknowledged the importance of communication, team work and mutual goals as crtitical elements in the implementation of any model of change. While the change was readily embraced in principle by all multidisciplinary teams, gaps in consultation processes with individual team members led to some inconsistency in how the process was operationalised. Apprehension experienced by some team members, especially the challenge of Day 0 mobility, led to inflexibility in how the workload was distributed, especially when unexpected challenges (prolonged physiotherapy leave) arose:

*“And some people on the ward did try, there were some attempts for day zero. But it wasn’t consistently done. And so I guess it’s still a question mark” (Participant 5).*

As such, Day 0 mobility was unachievavble for a considerable period of the implementation. In contrast, the alternate agreement of 4 episodes of mobility over a 2 day period was more readily embraced by members delivering the care, leading to greater consistency in how team members approached and supported this objective:

*“It helps out quite a bit especially the nurses as well, so if the nurse is pretty on board with it then they will set up everything beforehand and like everything is more efficient. They’ll set up the pain relief beforehand as well, before we came” (Participant 6).*

## Discussion

This novel study, examining a new mobilisation protocol in isolation with an embedded qualitative component, demonstrates that introduction of an early mobilisation protocol is associated with a modest reduction in LOS at a high-volume hospital whilst accounting for other factors. We interpret the association as likely to be real given the markers of protocol adherence (i.e. mobilisation statistics) indicate the protocol was followed (though not fully), the ‘time to clearance’ by allied health staff corroborated a reduction in LOS, and other care processes that could contribute to changes in mobilisation timing or LOS remained stable across time. We also acknowledge that our intervention was anchored on attempts to mobilise rather than achievement of a set distance or time spent mobilising. A more demanding intervention may have been more effective, but would have likely imposed greater burden on the staff. A reduction in the use of nerve blocks may increase the number of patients who could achieve early mobilisation. Importantly, the protocol was applied unreservedly allowing us to determine that early mobilisation is a strategy that can be trialled safely regardless of specific patient factors such as increasing obesity, increasing age, and language barriers. That said, whilst the application of the protocol need not be selective, we acknowledge that the association between early mobilisation and LOS is influenced by these same factors.

Many previous studies have procured more impressive reductions in LOS – up to 1.8 days as per a recent systematic review [6] - but we attribute this to the fact that early mobilisation has often been introduced as part of multiple changes that streamline care. It stands to reason then, that a single intervention, such as trialled here, would have a smaller effect. This not withstanding, equally modest or no reductions in LOS have been observed previously [14, 21, 33] and we interpret this knowledge as evidence that ‘context’ (eg. site) likely matters when implementing evidence into practice.

Our qualitative study has highlighted the importance of demarcating roles when introducing new interventions given that lack of clarity can undermine uptake. The audited data suggested nursing staff were less involved in the implementation, but the interviews revealed a different picture – a willingness to assist, but a struggle with the challenge of autonomous decision making, especially one that carries a perceived increased risk to patients. Together with the audited data, qualitative insights also reinforced the problem with afternoon surgeries especially in the absence of evening physiotherapy for commencing mobilisation on Day 0 or split distribution of Day 0 mobilisation across physiotherapy and nursing teams. We note that an earlier study was more successful in increasing the proportion who mobilised Day 0 in part due to introducing a swing shift from 11 a.m. to 8 p.m. in order to include those who underwent afternoon surgeries better [34]. Such a shift was not a feasible option at our facility. In addition, staff perceptions corroborated the study team’s concern (in the planning stage) about the need for adequate staffing to achieve the four mobilisation occasions by end Day 2. For staff, the challenges in attempting to implement a change in the absence of substantial increases in resources meant they questioned the value of early mobilisation when it was not successful for all patients. Such insight is not typically evident in quantitative explorations and yet is helpful for determining sustainability.

In addition to applying an early mobilisation protocol in isolation and the indepth qualitative investigation, other strengths of our study include the prospective design, the relatively large sample size, consecutive recruitment of patients, the monitoring of care processes that may confound or modify the associations, the collection of compliance data, comprehensive collection of patient and care covariates, and the capture of both early and longer-term outcomes. Limitations of our study include lack of adjustment for multiple testing and inability to include TUG – a variable shown to predict LOS [25] - in the adjusted modelling given it was not collected routinely. We also should note that the mobilisation profile underestimates the true number of attempts to mobilise patients early because we could only audit successful attempts as not all attempts were recorded if they were not successful. Finally, the unplanned decrease in physiotherapy cover was perceived to have greatly undermined the implementation, but we continued with the study in order to document implementation of the evidence under real-world conditions.

## Conclusion

Small reductions in LOS are possible following the introduction of an early mobilisation protocol in isolation following TKA or THA without compromising care, although staff burden is increased, likely undermining both sustainability and the magnitude of the reduction.

## Supplementary Information

**Additional file 1:** **Table 1A.** Variables included in regression modelling.

**Additional file 2.**

## Data Availability

The datasets supporting the conclusions of this article are available from the corresponding author on reasonable request.

## Abbreviations

ASA: American Society of Anesthesiologists
BMI: Body Mass Index
EQ-5D: EuroQOL 5 dimension
EQVAS: EuroQOL Visual Analogue Scale
ERAS: Enhanced Recovery After Surgery
FET: Fisher’s Exact Test
HCP: Health Care Professional
HDU: High Dependency Unit
ICU: Intensive Care Unit
IQR: Interquartile Range
LOS: Length of Stay
LTFU: Lost To Follow Up
MDT: Multidisciplinary Team
MET: Medical Emergency Team
NA: Not Available
SAS: Statistical Analysis Software
SD: Standard Deviation
SPSS: Statistical Product and Service Solutions
THA: Total Hip Arthroplasty
TKA: Total Knee Arthroplasty
TUG: Timed Up and Go
χ2: Chi Squared test
