# Supplementary material for: Implementing early mobilisation after knee or hip arthroplasty to reduce length of stay: a quality improvement study with embedded qualitative component
Source: BMC Musculoskelet Disord. 2020 Nov 20;21:765. doi: 10.1186/s12891-020-03780-7 (PMC7678277; doi:10.1186/s12891-020-03780-7)
Supplement: Supplementary file 1 — Additional file 1: Table 1A. Variables included in regression modelling. [file 12891_2020_3780_MOESM1_ESM.docx]

**Additional file 1**

**Table 1A. Variables included in regression modelling**

|  | Type | Unit and or reference |
| --- | --- | --- |
| Intervention period | Nominal | Historical |
| Age | Scalar | years |
| Body mass index | Scalar | kg/m^2^ |
| Sex | Nominal | Female |
| Primary diagnosis osteoarthritis | Nominal | Other |
| American Society of Anesthesiologists Grade (ASA) 3 or 4 | Nominal | ASA 1 or 2 |
| Interpreter required | Nominal | No |
| TKA | Nominal | THA |
| Other lower limb or back pain | Nominal | Nil |
| Unilateral or bilateral | Nominal | Bilateral |
| Day of surgery | Nominal (5 levels) | Monday |
| Morning surgery | Nominal | Before 12pm; after 12 pm (reference) |
| ICU or HDU admission | Nominal | Nil = reference |
| Acute complication | Nominal | Nil = reference  Complications include –  Major – venous thromboembolism, death, revision surgery, acute myocardial infarction, deep infection, fall with injury  Minor – cardiac arrythmia, fall without injury |
| Peripheral nerve block | Nominal | Nil |
