# Supplementary material for: Implementing early mobilisation after knee or hip arthroplasty to reduce length of stay: a quality improvement study with embedded qualitative component
Source: BMC Musculoskelet Disord. 2020 Nov 20;21:765. doi: 10.1186/s12891-020-03780-7 (PMC7678277; doi:10.1186/s12891-020-03780-7)
Supplement: Supplementary file 2 — Additional file 2. [file 12891_2020_3780_MOESM2_ESM.docx]

**Topic Guide**

| **Aim** | **Proposed questions** |
| --- | --- |
| **Care provided**  Aim: To explore the individual’s overall impression of the ERAS protocol | **Q1** Considering your experience with the implementation of the Early Rehabilitation after Surgery (ERAS) protocol on the orthopaedic ward over the last year, how would you describe the care (or patient experience) provided to patients undergoing total hip replacement or total knee replacement?   - Prompt: Reflecting on your past experiences with the post-operative management of patients undergoing THA or TKA, what do you see as the key differences or similarities in care (or patient experience) under the ERAS protocol?   **Q2** As part of the ERAS protocol patients are management by a team of health professionals. What has been your experience of the care provided by other MDT members under the ERAS protocol?   - Prompt: Reflecting on your past experiences with post-operative management are there any key differences in the operation of the MDT? |
| **Patient outcomes under an ERAS protocol**  Aim: To understand staff perspectives on patient outcomes under the ERAS protocol and how this compares to previous models of service delivery | **Q3:** In your experience has implementing the ERAS protocol on ____ ward made a difference to patient outcomes? In what ways  **Q4**: What do you see as the potential benefits to patients in an ERAS program based on your experience over the past 12 months?  Prompts:   - Are there any patient groups you see as likely to benefit most under an ERAS protocol? - What patient characteristics do you consider to be associated with superior outcomes under an ERAS protocol and why?   **Q5:** Based on your experience with ERAS, what do you consider as the potential risks to patients or adverse consequences patients may experience under an ERAS protocol compared to usual practice?   - Prompt: Are there any patient groups you see as vulnerable to complications under and ERAS protocol and why? - What patient characteristics do you consider to be associated with poorer outcomes under and ERAS protocol? |
| **Patient characteristics and ERAS**  Aim: To understand staff reasoning around patient suitability of the ERAS protocol | **Q6**: Some people feel that not all patients ‘fit standard care trajectories’ and are not suitable for protocols such as the ERAS protocol. What is your experience with the ERAS protocol and its suitability for all patients?  Prompt   - Are there some patients you think should be excluded from the ERAS protocol, for example based on co-morbidity, age, culture, language proficiency, etc?   **Q7** What patient characteristics make it challenging to implement the ERAS protocol or influence discharge destination or length of stay?  Prompt:   - In what way does patient characteristics such as age, expectations, ethnoculture, language proficiency, social supports and resources influence a patients success or otherwise with discharge home or within the recommended timeframe? |
| **Implementing ERAS**  Aim: To understand staff perspectives on team or institutional factors that may have influenced the implementation of the ERAS protocol | **Q8:** Are there any challenges you have personally experienced implementing the ERAS protocol with patients over the last 12 months?  **Q9** Some people feel that success with a model such as ERAS depends on the team and hospital implementing the changes. What are your thoughts?  Prompt:   - What are the team or institutional factors that facilitated early rehabilitation? - What are the team or institutional factors that hindered early rehabilitation? |
| **Recommendations**  Explore recommendations to improve patient or staff experiences | **Q10:** This concludes our main questions. Do you have any additional comments or recommendations arising from your experience with the ERAS protocol that you wish to share?   - Prompt: Are there any recommendations you have for how to optimise the patient and/or team experience implementing a protocol such as this long term? |

Modelled on previous literature related to this topic:

Allvin, R., M. Ehnfors, N. Rawal and E. Idvall (2008). "Experiences of the postoperative recovery process: an interview study." Open Nurs J **2**: 1-7.

Berthelsen, C. B. and K. Frederiksen (2017). "Orchestrating care through the fast-track perspective: A qualitative content analysis of the provision of individualised nursing care in orthopaedic fast-track programmes." Int J Orthop Trauma Nurs **24**: 40-49.

Brown, C. J., B. R. Williams, L. L. Woodby, L. L. Davis and R. M. Allman (2007). "Barriers to mobility during hospitalization from the perspectives of older patients and their nurses and physicians." Journal of Hospital Medicine **2**(5): 305-313.

Brown, D. and A. Xhaja (2018). "Nursing Perspectives on Enhanced Recovery After Surgery." Surgical Clinics of North America **98**(6): 1211-1221.

Cohen, R. and R. Gooberman-Hill (2019). "Staff experiences of enhanced recovery after surgery: systematic review of qualitative studies." BMJ Open **9**(2): e022259.
